# Shifts in soil and plant functional diversity along an altitudinal gradient in the French Alps

**DOI:** 10.1186/s13104-021-05468-0

**Published:** 2021-02-08

**Authors:** Alexia Stokes, Guillermo Angeles, Fabien Anthelme, Eduardo Aranda-Delgado, Isabelle Barois, Manon Bounous, Nereyda Cruz-Maldonado, Thibaud Decaëns, Stéphane Fourtier, Grégoire T. Freschet, Quentin Gabriac, Daniel Hernández-Cáceres, Leonor Jiménez, Jing Ma, Zhun Mao, Beatriz Eugenia Marín-Castro, Luis Merino-Martín, Awaz Mohamed, Christian Piedallu, Carlos Pimentel-Reyes, Hans Reijnen, Frédérique Reverchon, Hervé Rey, Lavinia Selli, Christina Desireé Siebe-Grabach, Katrin Sieron, Monique Weemstra, Catherine Roumet

**Affiliations:** 1 AMAP, Univ Montpellier, INRAE, IRD, CIRAD, CNRS, 34000 Montpellier, France; 2 Red de Ecología Funcional, Instituto de Ecología, A.C., Xalapa, Veracruz, Mexico; 3 Terranova Lombricultores, Coatepec, Veracruz, Mexico; 4 CEFE, Univ Montpellier, CNRS, EPHE, IRD, Univ Paul Valéry Montpellier 3, Montpellier, France; 5 Station d’Ecologie Théorique et Expérimentale, CNRS, 09200 Moulis, France; 6 Low Carbon Energy Institute, China University of Mining and Technology, Xuzhou, 221008 China; 7 Laboratorio de Edafología Ambiental, Instituto de Geología, Universidad Nacional Autónoma de México, Mexico City, Mexico; 8 Université de Lorraine, AgroParisTech, INRAE, UMR Silva, Nancy, France; 9 Red de Estudios Moleculares Avanzados, Instituto de Ecología, A.C., Pátzcuaro, Michoacán, Mexico; 10 Laboratorio de Edafología Ambiental, Instituto de Geología, Universidad Nacional Autónoma de México, Mexico City, Mexico; 11 Centro de Ciencias de la Tierra, Universidad Veracruzana, 91090 Xalapa, Veracruz, Mexico; 12 Departamento de Biología y Geología, Física y Química inorg´anica, ESCET, Universidad Rey Juan Carlos, C/Tulip´an s/n, M´ostoles, 28933 Madrid, Spain

**Keywords:** Aggregate stability, Alpine ecosystems, Carbon, Community, Elevation, Environmental gradient, Infiltration, Macroinvertebrates, Microorganisms, Plant community traits, Soil biophysical properties

## Abstract

**Objectives:**

Altitude integrates changes in environmental conditions that determine shifts in vegetation, including temperature, precipitation, solar radiation and edaphogenetic processes. In turn, vegetation alters soil biophysical properties through litter input, root growth, microbial and macrofaunal interactions. The belowground traits of plant communities modify soil processes in different ways, but it is not known how root traits influence soil biota at the community level. We collected data to investigate how elevation affects belowground community traits and soil microbial and faunal communities. This dataset comprises data from a temperate climate in France and a twin study was performed in a tropical zone in Mexico.

**Data description:**

The paper describes soil physical and chemical properties, climatic variables, plant community composition and species abundance, plant community traits, soil microbial functional diversity and macrofaunal abundance and diversity. Data are provided for six elevations (1400–2400 m) ranging from montane forest to alpine prairie. We focused on soil biophysical properties beneath three dominant plant species that structure local vegetation. These data are useful for understanding how shifts in vegetation communities affect belowground processes, such as water infiltration, soil aggregation and carbon storage. Data will also help researchers understand how plant communities adjust to a changing climate/environment.

## Objective

Elevational gradients can be used as a space-for-time substitution to provide insights into the response of communities to climatic changes and the impact on the local environment [1, 2]. In particular, altitude integrates changes in the diverse conditions that determine soil biophysical properties [3], including temperature, soil moisture, solar radiation and input from vegetation [4]. In an attempt to disentangle the mechanisms through which vegetation affects soil biotic and abiotic processes at different elevations, we collated data on the biophysical environment, soil physico-chemical properties, plant community traits, microbial functional diversity and soil macroinvertebrate indicators.

Soil biophysical properties are heavily influenced by input rates and decomposability of organic matter, transport to deeper soil horizons and physical protection in aggregate complexes. Therefore, our dataset includes data from infiltration experiments in the field and the measurement of soil aggregate stability at different depths. Our data also allow researchers to explore the impact of plant communities and soil macroinvertebrates on soil properties and vice-versa. With a particular focus on three plant species that structure local vegetation communities, belowground community-level traits were measured to determine how plant community composition impacts soil biophysics and microbial activity. To our knowledge, this dataset represents the largest freely available collection of data on plant traits and soil variables along a 1000 m elevational gradient in a temperate climate. Methods for each measurement are provided in the data files and supplementary materials (Table 1).

**Table 1:** Overview of data files/data sets

Label	Name of data file/data set	File type (file extension)	Data repository and identifier (DOI or accession number)
Data file 1	Plots and map	MS Excel file (.xlsx)	Portail Data INRAE. https://doi.org/10.15454/E3SAPW (2020) [15]
Data file 2	Description of soil profiles	MS Excel file (.xlsx)	Portail Data INRAE. https://doi.org/10.15454/ZTCHEG (2020) [16]
Data file 3	Plot climate interpolated base	MS Excel file (.xlsx)	Portail Data INRAE. https://doi.org/10.15454/ZDODW2 (2020) [17]
Data file 4	Soil water potential 2018–2019	MS Excel file (.xlsx)	Portail Data INRAE. https://doi.org/10.15454/PJKD06 (2020) [18]
Data file 5	Plot vegetation 20 × 20 m	MS Excel file (.xlsx)	Portail Data INRAE. https://doi.org/10.15454/UGFNKE (2020) [19]
Data file 6	Stable state infiltration	MS Excel file (.xlsx)	Portail Data INRAE. https://doi.org/10.15454/PWWMYV (2020) [20]
Data file 7	Monolith aggregate stability	MS Excel file (.xlsx)	Portail Data INRAE. https://doi.org/10.15454/UOU4TO (2020) [21]
Data file 8	Monolith 1 × 1 m vegetation	MS Excel file (.xlsx)	Portail Data INRAE. https://doi.org/10.15454/FZ7RA7 (2020) [22]
Data file 9	Ecopics soil analysis	MS Excel file (.xlsx)	Portail Data INRAE. https://doi.org/10.15454/B3FP79 (2020) [23]
Data file 10	Ecopics aboveground biomass	MS Excel file (.xlsx)	Portail Data INRAE. https://doi.org/10.15454/V70ZST (2020) [24]
Data file 11	Ecopics community level physiological profiles	MS Excel file (.xlsx)	Portail Data INRAE. https://doi.org/10.15454/KFBNR8 (2020) [25]
Data file 12	Monolith earthworms	MS Excel file (.xlsx)	Portail Data INRAE. https://doi.org/10.15454/UBFC6D (2020) [26]
Data file 13	Ecopics monolith root traits	MS Excel file (.xlsx)	Portail Data INRAE. https://doi.org/10.15454/RARBD1 (2020) [27]

## Data description

We present data collected in 2018, at six altitudes along an elevational gradient (1400–2400 m), at Massif de Belledonne, France (Table 1, file 1, [5]). Bedrock was composed of Variscan metamorphic rocks and ophiolitic complexes. Soils were umbrisols and cambisols and laminar erosion was present (Table 1, file 2, [6]). Climatic data (Table 1, file 3) were estimated over 2004–2014, using the Aurelhy model [7]. Soil water potential and temperature were measured at each altitude for two growing seasons (2018–2019, Table 1, file 4).

Five 400 m^2^ plots were chosen at each altitude, so that two or three dominant and community-structuring species were present: *Picea abies* (L.) H. Karst, *Vaccinium myrtillus* L. and *Juniperus communis* L. A botanical survey was performed in the plot (Table 1, file 5) and one adult individual of each structuring species was selected. At the limit of the individual’s canopy on the downslope side, infiltration tests were performed to estimate water flow through soil [8] and hydraulic conductivity of the quasi-steady phase was calculated (Table 1, file 6). Soil samples were collected for an overall description of soil type per horizon (Table 1, file 2) and aggregate stability measurements (Table 1, file 7 [9]).

To investigate the relationships between soil biophysical properties and vegetation, plant community composition was measured in a 1.0 m^2^ subplot within each plot and close to each structuring species (Table 1, file 8). Rhizospheric soil attached to fine roots of the three structuring plant species was collected. A soil monolith (0.25 m × 0.25 m × 0.15 m depth) was excavated within each subplot (n = 70). Above the monolith, litter layer thickness (Table 1, file 9) and aboveground biomass per species were measured (Table 1, file 10).

Soil texture, cationic exchange capacity, pH, organic carbon, nitrogen content, nitrate and ammonium were determined on pooled soil samples harvested within each monolith (Table 1, file 9). The MicroResp™ system [10] was used to characterize microbial activity and functional diversity on air-dried bulk and rhizospheric samples, through the community level physiological profiles of the soil microbial communities (Table 1, file 11).

Soil macroinvertebrates were hand sorted from each monolith and fixed in 100% ethanol. Macroinvertebrates were identified at the order level, except for earthworms for which morphological diagnoses were combined with DNA barcoding to obtain species level assignations. Invertebrates within each taxa were counted and weighed (Table 1, file 12, [11]).

Roots were hand sorted, washed and sorted into categories according to their diameter and functionality: rhizomes, very coarse roots with diameter > 5 mm, coarse roots with diameter 2–5 mm and fine roots (< 2 mm). Fine roots were separated into absorptive and transport roots [12]. Two subsamples of roots < 5 mm were scanned and analysed using Winrhizo Pro (Regent Instruments, Canada). Several root traits were measured, including root length density and mass, diameter, specific root length and tissue density (Table 1, file 13, [13]. Chemical traits were measured on absorptive and transport roots (Table 1, file 13), including nitrogen, carbon content and hydrosoluble compounds, hemicellulose, cellulose and lignin content [14].

## Limitations

Although this dataset comprises a large number of field and laboratory data collated from 70 monoliths in 30 plots, long-term climatic data were not available for each of the six altitudinal levels. Therefore we used the Aurelhy model, that estimates climatic data to a resolution of 1 km [7]. As some altitudinal bands were located within 1 km of each other, data will be the same for those plots. Also, datasets refer specifically to three dominant and community-structuring plant species, limiting generalization at larger scales.

## Data Availability

The data described in this Data note can be freely and openly accessed on Portail Data INRAE under https://data.inrae.fr/dataverse/ecopics. Please see Table 1 for details and links to the data. Supplementary material can be accessed at: https://doi.org/10.15454/1S15ZS.
